# A generic cross-seeding approach to protein crystallization

**DOI:** 10.1107/S1600576725000457

**Published:** 2025-02-17

**Authors:** Ido Caspy, Shan Tang, Dom Bellini, Fabrice Gorrec

**Affiliations:** a MRC Laboratory of Molecular Biology, Francis Crick Avenue, Cambridge Biomedical Campus, CambridgeCB2 0QH, United Kingdom; b Division of Life Sciences and Medicine University of Science and Technology of China Hefei Anhui230001 People’s Republic of China; Oak Ridge National Laboratory, USA; North Carolina State University, USA

**Keywords:** X-ray crystallography, macromolecular crystallization, protein crystals, nucleation, cross-seeding

## Abstract

Obtaining diffraction-quality crystals is often the rate-limiting step during structure determination of biological macromolecules by X-ray crystallography. To address this problem, we investigated a cross-seeding approach with a mixture integrating a heterogeneous set of protein crystal fragments to be used as generic seeds. The fragments are nanometre-sized templates chosen to promote crystal nucleation of protein samples unrelated to the proteins forming the seeds. An atypical crystal form of the human serine hydrolase retinoblastoma binding protein 9 was obtained by adding the mixture to the protein sample before performing standard crystallization assays. The structure was solved by X-ray crystallography at 1.4 Å resolution. Follow-up experiments showed that crystal fragments of α-amylase were critical components in this particular result. The limitations and future applications of our experimental developments are discussed.

## Abstract 2

A mixture integrating a wide variety of protein crystal fragments was used to obtain a new crystal structure of the human retinoblastoma binding protein 9.

## 1. Introduction

In recent years, electron cryomicroscopy (cryo-EM) (Chua *et al.*, 2022 ▸; McMullan *et al.*, 2023 ▸) used in combination with *AlphaFold* (Jumper *et al.*, 2021 ▸) has become the leading approach for determining novel structures of large macromolecular complexes. In parallel, X-ray crystallography remains a powerful technique in drug discovery, enabling us to determine highly accurate structures of small- and medium-sized proteins (*i.e.* proteins consisting of several dozen to hundreds of amino acids) with small-molecule ligands (Käck & Sjögren, 2025 ▸). This is illustrated by the continued growth of crystal structures in the Protein Bata Bank (PDB) (https://www.rcsb.org/stats/growth/growth-xray). Other macromol­ecular crystal diffraction techniques further enhance our understanding of biological mechanisms and support structure-based drug design, such as neutron crystallography, time-resolved crystallography with X-ray free-electron lasers (XFELs) and microcrystal electron diffraction (microED). Neutron crystallography, in addition to the mapping of hydrogen atoms, enables the very accurate mapping of subtle interactions of multi-component systems (Meilleur, 2020 ▸). Time-resolved X-ray crystallography is mainly employed to study ultra-fast enzyme dynamics, *e.g.* in light-driven reactions (Orville *et al.*, 2024 ▸). A main advantage of XFELs and microED is to bypass the requirement of growing the relatively large crystals that are required for more conventional home-source or most synchrotron X-ray diffraction techniques (Nannenga & Gonen, 2019 ▸; Nanev *et al.*, 2023 ▸).

A fundamental problem shared by all crystal diffraction techniques is that the yield of diffraction-quality crystals is typically very low. Crystallization-governing properties, notably sample stability, solubility and ideally the absence of different conformations, are influenced by a very large number of factors (McPherson, 2017 ▸). Since each sample of biological macromolecules (proteins, DNA, RNA, small molecules and their complexes) has different and specific properties (Derewenda, 2010 ▸; Ferreira & Castro, 2023 ▸), it is unlikely that crystallization conditions can be predicted except in very well characterized cases. As a result, researchers must normally approach crystallization as a stochastic process, which requires a vast number of automated trials to screen empirically for suitable conditions (Gorrec & Löwe, 2018 ▸; Beale *et al.*, 2020 ▸; Lynch *et al.*, 2023 ▸). Once initial diffraction-quality crystals are obtained, optimization work is also often required. For example, producing different crystal forms can be critical for successful structure determination (Metz *et al.*, 2021 ▸) and applications to drug discovery require the best possible crystals (Vera *et al.*, 2013 ▸).

Although crystallization experiments have been miniaturized to nanolitre-scale drops, preparing enough sample for crystallization can be challenging and often results in limited material for extensive screening. This issue is especially pertinent these days, because cryo-EM requires much less material and therefore most projects no longer require large amounts of sample.

Seeding is a widely used and powerful method for improving protein crystallization (Stura & Wilson, 1990 ▸; Bergfors, 2003 ▸; Stura, 2013 ▸). Protein crystals obtained through screening, but of insufficient size, quantity or quality, are added to subsequent crystallization trials to lower the thermodynamic and kinetic barriers inherent in the various stages of crystal growth, primarily during nucleation (García-Ruiz, 2003 ▸; Houben *et al.*, 2020 ▸; Vekilov, 2016 ▸). Introducing nucleation seeds into crystallization setups allows crystals to grow during the earlier stages of experiments, when undesirable side effects such as precipitation and aggregation are less prevalent (Luft & DeTitta, 1999 ▸; Chayen & Saridakis, 2008 ▸). In addition, seeding is highly amenable to miniaturized and automated protocols, which are now standardized (D’Arcy *et al.*, 2007 ▸; Thakur *et al.*, 2007 ▸). The most common and efficient approach to seeding is a form of homoepitaxy, which uses seeds made from the same protein as the one intended for crystallization, which also normally leads to the same crystal form.

When there are no crystals resulting from the initial crystallization screening, crystals from homologous samples of the target protein can be used to prepare seeds. These seeds may facilitate a form of heteroepitaxial nucleation, also known as cross-seeding. Two problems make cross-seeding difficult in practice. First, crystals of homologous samples are not always available. Second, the requirements of successful cross-seeding are difficult to predict, particularly the required degree of similarity between the protein sequences and structures of two samples to positively influence the crystallization of one another. Approaches to cross-seeding usually involve closely related protein variants (Islam & Kuroda, 2017 ▸) or at least the same class of proteins (Abuhammad *et al.*, 2017 ▸). Alternatively, and to circumvent the need for initial protein crystals made of homologous proteins, different materials have also been investigated as nucleation agents in protein crystallization experiments, such as synthetic crystalline and porous polymers (Sugahara *et al.*, 2008 ▸), polystyrene microspheres (Guo *et al.*, 2014 ▸), and functionalized carbon nanoparticles (Govada *et al.*, 2016 ▸). The magnitude of the challenge of macromolecular crystallization tends to stimulate even bolder developments. For example, it has been observed several times that natural fibres such as hair can be useful and cost-efficient nucleation agents (D’Arcy *et al.*, 2003 ▸; Georgieva *et al.*, 2007 ▸).

If nucleation is a largely stochastic process, it is reasonable to think that a mix of seeds prepared with a broad variety of protein samples will increase the likelihood of promoting specific interactions that ultimately lead to crystal lattice formation with any given protein of interest (Shaw Stewart *et al.*, 2011 ▸; D’Arcy *et al.*, 2014 ▸; Yan *et al.*, 2018 ▸). This strategy is the basis of the developments of our generic cross-seeding approach described here. The main component is a mixture that integrates crystal fragments prepared from 12 unrelated commercially available proteins (called ‘host proteins’). The form and quality of the crystals made from the host proteins were first assessed by X-ray crystallography. The diffraction-quality crystals were then fragmented with high-speed oscillation mixing. The fragmentation process was characterized using cryo-EM, producing high-definition images of streptavidin crystal fragments.

The non-uniformly sized and shaped protein crystal fragments – made from proteins of a highly diverse nature – are at the centre of our efforts to enhance the probability of generic cross-seeding. An important consideration was the stability of seeds during the cross-seeding trials. To ensure this, the crystallization of the host proteins, their fragmentation and the subsequent cross-seeding were conducted using MORPHEUS crystallization solutions and conditions, which formulation integrates highly compatible PEG-based precipitant mixes, buffer systems (pH range 6.5–8.5) and stabilizing additives (Gorrec, 2009 ▸; Gorrec & Bellini, 2022 ▸).

To demonstrate applicability, the generic cross-seeding mixture was simply added to a sample of the human retinoblastoma binding protein 9 (RBBP9) (Vorobiev *et al.*, 2009 ▸; Tang *et al.*, 2022 ▸) before proceeding with crystallization. An atypical crystal form of RBBP9 was then obtained, and the corresponding structure determined to 1.4 Å resolution by X-ray crystallography. Further investigation revealed that the integration of fragments made of α-amylase into the mixture was essential in this case.

## 2. Materials and methods

### 2.1. Crystallization of the host proteins

The 12 host proteins (Table 1 ▸) were purchased as lyophilized powders, apart from Catalase, which was obtained in solution (Merck, see description accompanying Table S1 of the supporting information). The lyophilized proteins were gently mixed in their buffer (or only Milli-Q water, see column ‘Buffer’ in Table 1 ▸) and left to hydrate for 24 h at 4°C. The samples were mixed again and filtered (0.22 µm). The following crystallization experiments were performed as 48 repeats using vapour-diffusion sitting drops in MAXI plates (SWISSCI), set up on a Mosquito liquid handler (SPT Labtech) at 20°C. The reservoirs contained 200 µl of crystallization solutions from the MORPHEUS crystallization screen or MORPHEUS-FUSION screen (Molecular Dimensions). Different protein-to-solution ratios were used for a final drop volume of 1.5 µl. Plates were immediately sealed after setting up the crystallization drops with 3 inch-wide sealing tape (Crystal Clear, Hampton Research) and stored at 18°C. Plates were assessed regularly for up to 15 weeks using a Leica M205C stereomicroscope before X-ray data collection and cross-seeding mixture preparation. Images were taken on an M205C microscope (Leica) equipped with a CS505CU Kiralux camera (Thorlabs). More details on the crystallization of the host proteins can be found in the supporting information.

### 2.2. Preparation of the cross-seeding mixture

**Figure 1:**
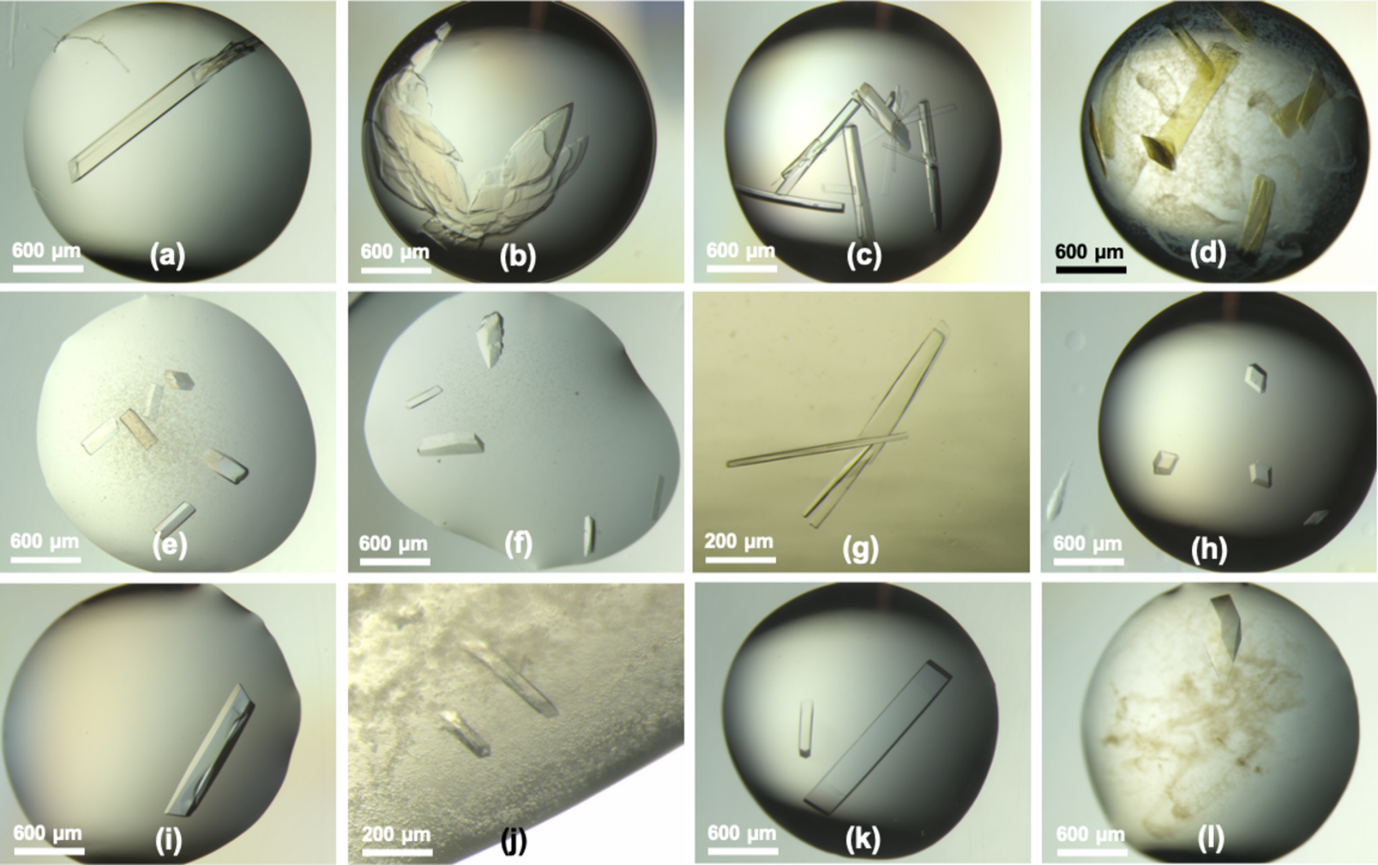
Light micrographs showing representative crystallization drops (*i.e.* hits) with diffraction-quality crystals of the 12 host proteins: (*a*) α-amylase, (*b*) albumin, (*c*) aprotinin, (*d*) catalase, (*e*) concanavalin A, (*f*) creatine kinase, (*g*) glutathione *S*-transferase, (*h*) insulin, (*i*) lysozyme, (*j*) pyruvate kinase, (*k*) streptavidin and (*l*) thaumatin.

The stabilizing solution was formulated with 24%(*v*/*v*) PEG 500 MME (polyethylene glycol monomethyl ether), 12%(*w*/*v*) PEG 20K (polyethylene glycol) and 0.1 *M* sodium–HEPES:MOPS [3-(*N*-morpholino)propanesulfonic acid] buffer system, titrated to pH 7.5. Three representative drops (Fig. 1 ▸) for each host protein were combined in a 1.5 ml tube. This was carried out at room temperature (18°C) on the stage of a Leica M205C microscope, with a MICROMAN P10 positive displacement pipette, fitted with long and flexible capillary piston tips (Gilson, F148412). The amount of contaminating precipitate was reduced with the repeated addition and removal of stabilizing solution. The largest crystals were broken apart using the tip end, after which most of the drop volume (1.5 µl) was aspirated and transferred to the bottom of the tube. Then 1–2 µl of stabilizing solution was added to the well, aspirated and transferred to the tube. The resulting volume of stabilizing solution combining all crystals was 240 µl. After gentle mixing of the viscous solution with a 1000 µl pipette, the mixture was split into three reservoirs of a 96-well MRC plate (SWISSCI) to enable further fragmentation of the crystals with five cycles of 2 min on a high-speed oscillation mixer (MXone, SPT Labtech). The three 80 µl samples in the reservoirs were re-combined gently in a 1.5 ml tube, to spin the fragments to the bottom of the tube for 10 min at 500 rev min^−1^ (centrifuge 5424, Eppendorf). Two-thirds of the supernatant (∼140 µl) was then gently aspirated from the top and discarded. The remaining 80 µl was gently aspirated and constituted the final preparation of the cross-seeding mixture. The resulting ∼20 µl at the bottom of the tube containing the largest fragments was also discarded. Cross-seeding mixture aliquots of 2 µl were dispensed in 100 µl PCR tubes with the positive displacement pipette and stored at −20°C until further use.

### 2.3. Inspection of the crystal fragments by cryo-EM

Relatively large, elongated streptavidin crystals (300–1500 µm) were harvested from representative drops [Fig. 1 ▸(*k*)], as described in the previous section. The crystals were transferred to the reservoir of an MRC plate (80 µl of the crystallization condition in total) for fragmentation on a high-speed oscillation mixer (MXone, SPT Labtech), with two cycles of 2 min. The solution containing the fragments was transferred to a 200 µl PCR tube and underwent centrifugation for 5 min at 500  rev min^−1^ (centrifuge 5424, Eppendorf), then 40% of the supernatant (32 µl) was discarded. For grid preparations, 2.5 µl of the solution of streptavidin crystals, or the cross-seeding mixture [diluted 1:1 (*v*/*v*) with Milli-Q water], was applied to glow-discharged Quantifoil R 3.5/1 Cu/Rh 200 mesh cryo-EM grids. The grids were back-side blotted, supported by a Teflon pad replacing the blotting paper on the sample side. The grids were plunge-frozen into liquid ethane in a temperature-regulated cryostat device using a Vitrobot Mark IV (Thermo Fisher Scientific, TFS) at 100% relative humidity and a chamber temperature of 15°C (McDowall *et al.*, 1983 ▸; Russo *et al.*, 2016 ▸). Transmission electron micrographs of streptavidin crystal fragments were produced using a Glacios microscope (TFS) with a Falcon 3 direct electron detector (TFS) at a voltage of 200 kV with a total dose of 40 e^−^ Å^−2^ and a pixel size of 2.545 Å. The fragments in the final cross-seeding mixture were imaged using a Titan Krios G3 microscope (TFS), equipped with a Quantum energy filter (slit width 20 eV) and a K3 direct electron detector (Gatan) at a voltage of 300 kV with a total dose of 40 e^−^ Å^−2^ and a pixel size of 2.128 Å.

### 2.4. Crystallization experiments with RBBP9

RBBP9 (∼22 kDa) was expressed in *Escherichia coli* and prepared at 10 mg ml^−1^ as described elsewhere (Tang *et al.*, 2022 ▸). The crystallization experiments with the cross-seeding mixture were triplicated in MRC plates (SWISSCI), in which the reservoirs contained 80 µl of the 96 conditions from the MORPHEUS-FUSION screen (Gorrec & Bellini, 2022 ▸). Drops of 400 nl (200 nl protein and 200 nl crystallization solution) were set up on a Mosquito liquid handler (SPT Labtech) at 20°C. The same protocol was followed for the RBBP9 sample with added cross-seeding mixture or a stabilizing solution as a control. For this, 30 µl of protein sample was gently mixed with 2 µl of the mixture (*i.e.* seed to protein ratio 1:15). Hence, the total number of crystallization drops prepared was 864 (96 conditions × 3 variations of the protein sample × 3 repeats). To prepare the seeding solutions made from a single host protein, only one representative drop (Fig. 1 ▸) was diluted in 80 µl of the stabilizing solution in the reservoir of an MRC plate (SWISSCI) for fragmentation with two cycles of 2 min on a high-speed oscillation mixer (MXone, SPT Labtech). The drops were set up on a Mosquito, running an automated sparse matrix microseeding protocol (D’Arcy *et al.* 2017 ▸): 500 nl of protein sample, 50 nl of seeds and 500 nl of crystallization solution (*i.e.* seed to protein ratio 1:10) were mixed. Plates were assessed regularly over a period of two weeks using an M205C microscope (Leica) equipped with a CS505CU Kiralux camera (Thorlabs).

### 2.5. Crystal screening, structure determination and analysis

Crystals were harvested from the drops with CrystalCap HT loops (Hampton Research) and flash-frozen in liquid nitro­gen. Crystallographic data were collected on beamline I04 of Diamond (Harwell, UK) at cryogenic temperature and processed with *DIALS* (Winter *et al.*, 2018 ▸). The crystal structures were solved by molecular replacement with *Phaser* (McCoy *et al.*, 2007 ▸). Interactive atomic model building was performed with *Coot* (Emsley *et al.*, 2010 ▸), refinement with *REFMAC5* (Murshudov *et al.*, 1997 ▸) and *Phenix* (Liebschner *et al.*, 2019 ▸), and geometric model validation with *MolProbity* (Chen *et al.*, 2010 ▸). Images of crystal structures and densities of charges on protein surfaces were generated with *ChimeraX* (Goddard *et al.*, 2018 ▸).

## 3. Results and discussion

### 3.1. Crystal fragments of the host proteins integrated to the cross-seeding mixture

After initial screening for solubility and crystallization, diffraction-quality crystals were obtained for all 12 commercially available proteins (Fig. 1 ▸). Of the 12 crystals, eight showed optimal growth in conditions with the MORPHEUS mix of precipitants that integrate a 2:1 ratio of PEG 500 MME to PEG 20K (Table 1 ▸). In fact, the four other proteins could also be crystallized in conditions with this precipitant mix, but somewhat less efficiently. As a result, the PEG 500 MME:PEG 20K mix was selected as the main component for the solution to combine and store the seeds. In addition, this mix was anticipated to be compatible with most protein samples to be crystallized and the PEG-based MORPHEUS crystallization conditions.

Our set of host proteins integrates proteins with highly diverse functions and sizes (Table S1). Using the crystals we obtained for the host proteins, we solved their crystal structures (Table 2 ▸), interestingly yielding mostly structures in the space groups *P*2_1_2_1_2_1_ (4/12) and *P*2_1_ (2/12), as was also observed for much larger datasets in the PDB (Wukovitz & Yeates, 1995 ▸; Gaur, 2021 ▸).

**Figure 2:**
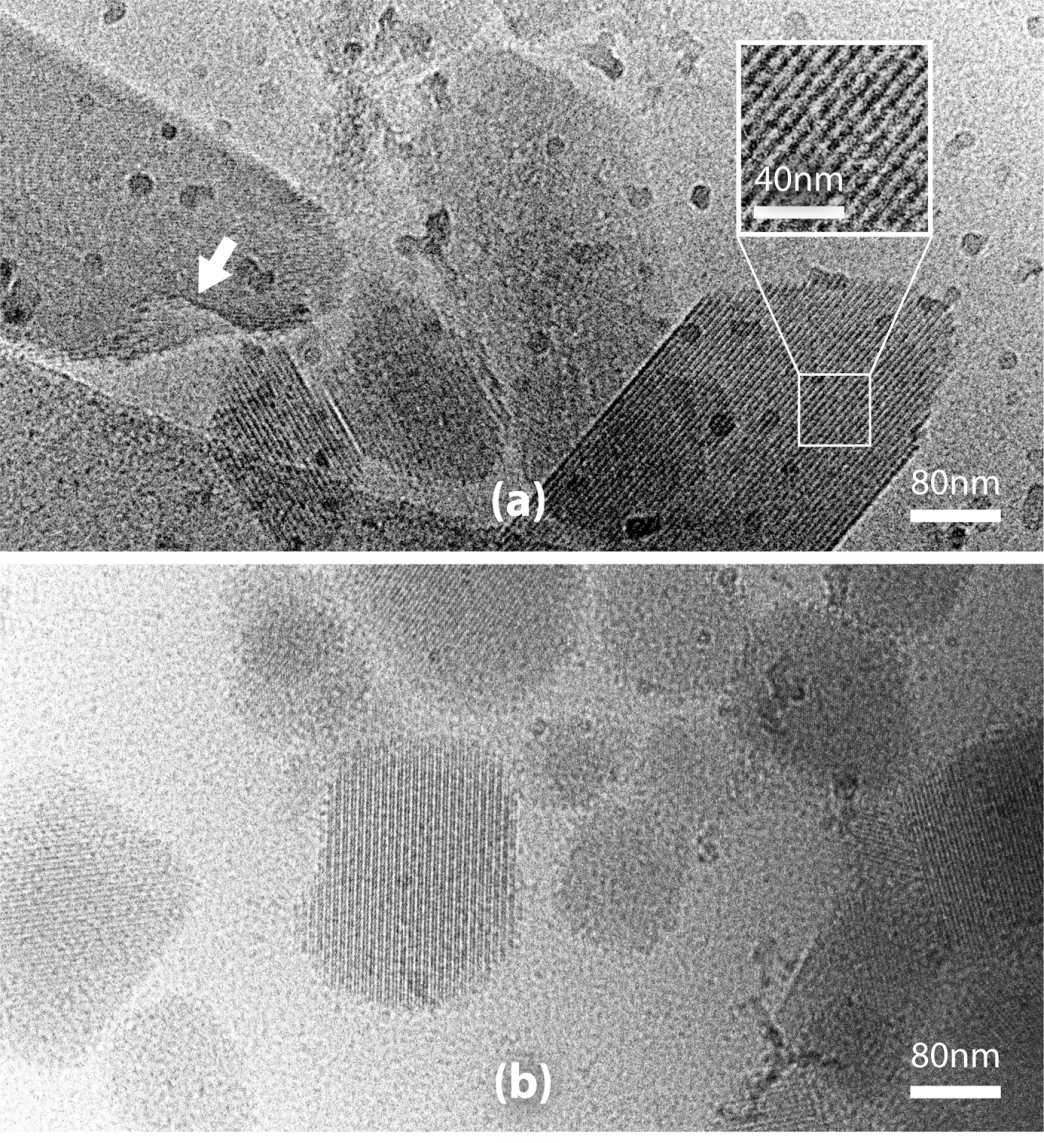
Transmission electron cryomicrographs of protein crystal fragments. (*a*) Fragments of streptavidin crystals. The arrow indicates a cuticle step edge. Inset: magnified view of a fragment showing its highly ordered structure. (*b*) Fragments of different protein crystals observed in the final cross-seeding mixture.

Preparations of crystal fragments were imaged with cryo-EM, initially to reveal the structural features of the fragments while optimizing the fragmentation, and later to enable quality control of the cross-seeding mixture. The aim was to produce nanometre-sized crystal fragments with a high degree of variance in their structures and exposed surfaces that could serve as potential nucleation templates. Fig. 2 ▸(*a*) shows crystal fragments generated from streptavidin crystals, displaying the expected highly ordered crystalline structure (Table 2 ▸). The fragmentation process generated different irregular morphologies and surface cavities, including cuticle step edges.

Screening the samples with cryo-EM presented well known challenges (Han *et al.*, 2023 ▸) and resulted in poor yields of useful images. This was particularly true when investigating the nature of the cross-seeding mixture. Although relatively low concentrations of PEG can be used for cryo-EM sample preparation (Rastegarpouyani *et al.*, 2023 ▸), working at high concentrations of PEGs was not amenable to cryo-EM imaging. However, images of the cross-seeding mixture at medium magnification allowed us to confirm that the seeds did not particularly tend to clump and provided a measurement of the size range of the seeds, which was between 60 and 150 nm [Fig. 2 ▸(*b*)].

The cross-seeding mixture was initially tested in homoepitaxial seeding assays. For this, the mixture was added to each of the host protein samples before setting up sitting drops with the crystallization condition used for the host protein (Table 1 ▸). These experiments resulted in substantial increases in nucleation sites compared with the controls without seeds (see Fig. S1 of the supporting information as an example).

Incorporating an even broader variety of proteins and crystals in the mixture would presumably increase the chances of interactions with the sample to be crystallized and potentially promote cross-seeding. Significant practical challenges could however curtail further development of the mixture, since each sample comes with its own problems when trying to produce large numbers of crystals (Deng *et al.*, 2004 ▸; Newman *et al.*, 2007 ▸; St John *et al.*, 2008 ▸; Niedzialkowska *et al.*, 2016 ▸). Also, the viscosity of the mixture and the risk of fragments interacting in some way will increase.

### 3.2. Crystallization of human retinoblastoma binding protein 9

**Figure 3:**
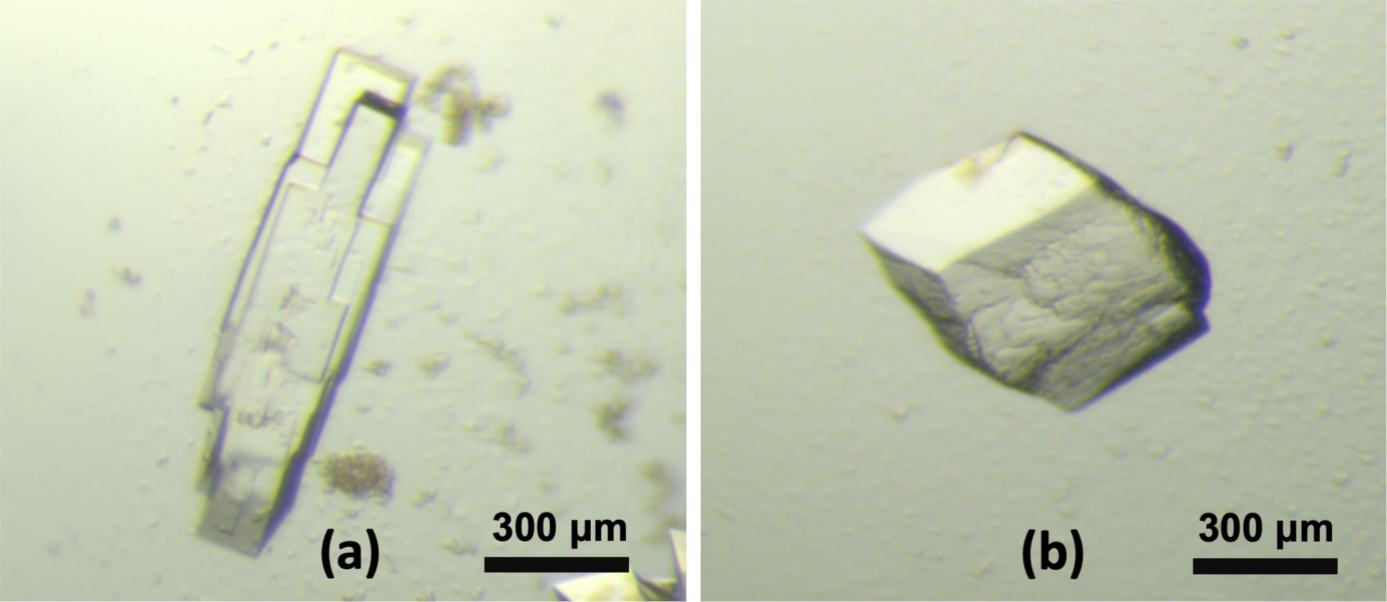
Light micrographs showing the two morphologies of RBBP9 crystals. (*a*) Rod-like crystals (space group *P*2_1_). (*b*) Crystal with almost equal dimensions in all directions (*P*2_1_2_1_2_1_).

The triplicated crystallization experiments resulted in 60 crystallization drops that were considered ‘hits’ (*i.e.* drops that contained crystals large enough to be fished out readily and later showed good diffraction; Table S2). Experiments with the cross-seeding mixture had a somewhat higher yield compared with experiments without: 25 with versus 19 without addition, and 16 hits with the addition of the stabilizing solution (although these numbers are not statistically significant). Most hits exhibited very similar looking crystals: bundles of more or less elongated rod-shaped structures, such as those grown in MORPHEUS-FUSION condition G8 [Fig. 3 ▸(*a*)]. After screening crystals from multiple hits without seeding mix added, isolating single rods when feasible, we determined the space group of the main crystal morphology as *P*2_1_, matching that reported in the literature for RBBP9 [PDB entries 2qs9 (Vorobiev *et al.*, 2009 ▸) and 7oex (Tang *et al.*, 2022 ▸)].

**Figure 4:**
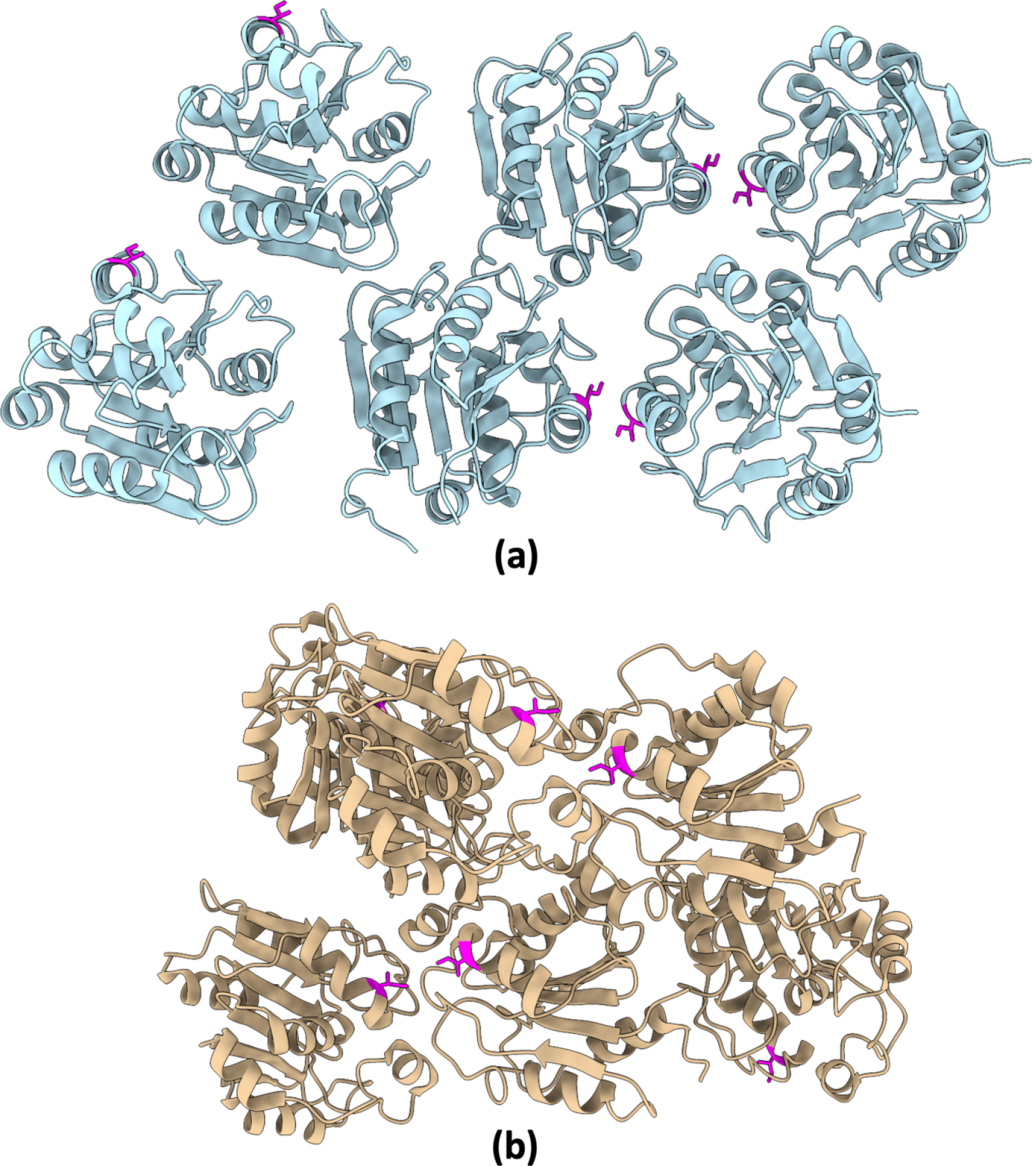
Ribbon representation of the crystal structures of RBBP9. (*a*) *P*2_1_ form (PDB entry 7oex) shown in light blue. (*b*) *P*2_1_2_1_2_1_ form (PDB entry 9fcr) shown in tan. Isoleucine 54 is shown in magenta in every asymmetric unit to better visualize the different packing arrangements in the two crystal forms.

A different crystal morphology was only observed when the seeding mixture was added to the sample in two screening conditions: MORPHEUS-FUSION D5 {12.5% *w*/*v* PEG 1000, 12.5% *w*/*v* PEG 3350, 12.5% *v*/*v* MPD [(*RS*)-2-methyl-2,4-pentanediol], 0.1 *M* MOPS/HEPES-Na [4-(2-hydroxyethyl)piperazine-1-ethanesulfonic acid], sodium salt pH 7.5, 20 m*M* amino acids, 20 m*M* monosaccharides} and F8 {12.5% *w*/*v* PEG 1000, 12.5% *w*/*v* PEG 3350, 12.5% *v*/*v* MPD, 0.1 *M* MES [2-(*N*-morpholino)ethanesulfonic acid]/imidazole pH 6.5, 1 m*M* alkalis, 0.5% *w*/*v* cryo-polyols}. These crystals were single and not elongated [Fig. 3 ▸(*b*)]. Crystallographic data analysis and structure determination of the crystals with this newly characterized morphology for the RBBP9 protein also revealed a different crystal lattice, as shown in Fig. 4 ▸ (space group *P*2_1_2_1_2_1_; PDB entry 9fcr; Table S3). However, no unexplained electron density was found that would indicate a cross-seeding fragment caused specific interactions leading to switch the crystal form.

To further investigate which component of the mixture altered the crystallization behaviour, 12 solutions containing seeds from each host protein were prepared separately. This time, to save RBBP9 sample, the mix and separate seeding solutions were added directly to the protein crystallization drops (D’Arcy *et al.* in 2007 ▸). In addition, screening was only done against the two conditions that produced the newly characterized crystal form. The atypical crystal morphology appeared in 2 of the 19 hits obtained (Table S4, Fig. S2). One hit was in condition D5, reproducing the initial result with the cross-seeding mixture; the other hit was in condition F8, when adding the seeds made of α-amylase [∼53 kDa, space group *I*222; PDB entry 7p4w (Gorrec & Bellini, 2022 ▸)]. Crystallographic data analysis and structure determination of the crystals confirmed again the two distinct molecular packings described above (Fig. 4 ▸).

Mechanisms of protein crystallization facilitated by cross-seeding are driven by the complex surface chemistry, charge and topography of the nucleation agent. These factors can manifest in different ways, depending on the nature of the interactions between the nucleation agent and the protein to be crystallized. When the surface of the seeds induces the stabilization of protein clusters, the local concentration on the seed may become high enough to promote crystal nucleation (Georgieva *et al.*, 2007 ▸; Tosi *et al.*, 2008 ▸; Shah *et al.*, 2012 ▸; Nanev *et al.*, 2021 ▸; Dunn *et al.*, 2023 ▸). It is reasonable to speculate that repetitive chemical features on the surface of the seeds, as they are crystal fragments, could have promoted an ordered adsorption of the guest protein molecules as building blocks required for nucleation (Van Driessche *et al.*, 2018 ▸).

However, we could not produce a model describing the possible mechanisms of cross-seeding that could have directly promoted the different crystal form of RBBP9 (space group *P*2_1_2_1_2_1_) with α-amylase fragments, for example by comparing their lattices. That is probably to be expected given the underlying complexities of the process (Sauter *et al.*, 2015 ▸; Van Driessche *et al.*, 2022 ▸). In fact, other speculations could be based on indirect effects of the seeds instead of cross-seeding. For example, α-amylase fragments could act as purifying agents, by mobilizing poorly folded RBBP9 molecules, which enabled the growth of a different crystal form.

Progress in macromolecular crystallography (Agirre *et al.*, 2023 ▸) and analysis of molecular interfaces (Krissinel & Henrick, 2007 ▸; Carugo *et al.*, 2017 ▸; Elez *et al.*, 2018 ▸; Porter *et al.*, 2019 ▸; Bryant *et al.*, 2022 ▸) combined with accurate, state-of-the-art predictions of protein–protein interactions with tools such as *AlphaFold-Multimer* (Evans *et al.*, 2022 ▸) are needed to facilitate the development of cross-seeding approaches and a better understanding of nucleation at the molecular level.

## 4. Conclusions

The potential usefulness of an approach to generic cross-seeding for protein crystallization was demonstrated and its limitations discussed. Our work included the development of a method for preparing seeds by high-speed mixing, generating a multitude of types of fragments. The mixture tested here is cost effective and suitable as an off-the-shelf solution that can simply be added to protein samples before proceeding with standard protein crystallization protocols. While testing of many more samples and conditions will be required to evaluate the full potential of our approach, and eventually visualize the mechanisms of cross-seeding, a bewildering array of other cross-seeding mixtures could be contemplated, for example with a set of hyper-stable engineered protein nanomaterials (Zhang *et al.*, 2020 ▸; Hsia *et al.*, 2021 ▸), or by gathering a wider variety of crystallization hits obtained in a protein crystallization facility (D’Arcy *et al.*, 2014 ▸).

## Supplementary Material

Supporting tables and figures. DOI: 10.1107/S1600576725000457/ei5125sup1.pdf

PDB reference: human serine hydrolase retinoblastoma binding protein 9, 9fcr

## Figures and Tables

**Table 1:** Crystallization solutions and conditions used for the 12 host proteins (*a*)–(*l*)

	Protein	MW (KDa)	Conc. (mg ml^−1^)	Buffer	Condition (sample:conditon ratio)	Formulation
(*a*)	α-Amylase	53	30	No addition (Milli-Q water only)	80% FUSION H11 (2:1)	16% *v*/*v* PEG 500 MME; 8% *w*/*v* PEG 20000; 64 m*M* polyamines; 80 m*M* buffer system 1 pH 6.5
(*b*)	Albumin	66.5	50	No addition (Milli-Q water only)	MORPHEUS H9 (2:1)	20% *v*/*v* PEG 500 MME; 10% *w*/*v* PEG 20000; 0.1 *M* amino acids; 0.1 *M* buffer system 3 pH 8.5
(*c*)	Aprotinin	6.5	45	75 m*M* citric acid pH 4.0	MORPHEUS C5 (1:1)	20% *v*/*v* PEG 500 MME; 10% *w*/*v* PEG 20000; 90 m*M* NPS mix; 0.1 = *M* buffer system 2 pH 7.5
(*d*)	Catalase	248	9	10% glycerol added, traces of thymol	MORPHEUS C1 (2:1)	20% *v*/*v* PEG 500 MME; 10% *w*/*v* PEG 20000; 90 m*M* NPS mix; 0.1 *M* buffer system 1 pH 6.5
(*e*)	Concanavalin A	104–112	10	10 m*M* Tris pH 8.0	FUSION D8 (2:1)	20% *v*/*v* glycerol; 10% *w*/*v* PEG 4000; 4 m*M* alkalis; 0.1 *M* monosaccharides 2; 0.1 *M* buffer system 3 pH 8.5
(*f*)	Creatine kinase	84	15	10% *v*/*v* MORPHEUS LiNaK mix	80% MORPHEUS F12 (1:1)	10% *v*/*v* MPD; 10% PEG 1000; 10% *w*/*v* PEG 3350; 0.10 *M* monosaccharides; 0.08 *M* buffer system 3 pH 8.5
(*g*)	Glutathione *S*-transferase	50	5	10% *v*/*v* MORPHEUS cholic acid mix	MORPHEUS G5 (1:1)	20% *v*/*v* PEG 500 MME; 10% *w*/*v* PEG 20000; 0.1 *M* carboxylic acids; 0.1 *M* buffer system 2 pH 7.5
(*h*)	Insulin	5.8	5	20% *v*/*v* MORPHEUS B11 (traces of zinc)	70% MORPHEUS C7 (2:1)	14% *v*/*v* glycerol; 7% *w*/*v* PEG 4000; 70 m*M* NPS mix; 70 m*M* buffer system 2 pH 7.5
(*i*)	Lysozyme	14.4	12.5	10% *v*/*v* MORPHEUS buffer system 1 pH 6.5	MORPHEUS G5 (2:1)	20% *v*/*v* PEG 500 MME; 10% *w*/*v* PEG 20000; 0.1 *M* carboxylic acids; 0.1 *M* buffer system 2 pH 7.5
(*j*)	Pyruvate kinase	237	25	No addition (Milli-Q water only)	MORPHEUS H2 (1:1)	20% *v*/*v* ethylene glycol; 10% *w*/*v* PEG 8000; 0.1 *M* amino acids; 0.1 *M* buffer system 1 pH 6.5
(*k*)	Streptavidin	53.6	9	10% MORPHEUS polyamine mix	MORPHEUS G5 (2:1)	20% *v*/*v* PEG 500 MME; 10% *w*/*v* PEG 20000; 0.1 *M* carboxylic acids; 0.1 *M* buffer system 2 pH 7.5
(*l*)	Thaumatin	22	20	5 m*M* disodium tartrate	MORPHEUS G9 (1:2)	20% *v*/*v* PEG 500 MME; 10% *w*/*v* PEG 20000; 0.1 *M* carboxylic acids; 0.1 *M* buffer system 3 pH 8.5

**Table 2:** Characterization of the diffraction-quality crystals (*a*)–(*l*) used to prepare the cross-seeding mixture

	Protein	Space group	Unit-cell parameters *a*, *b*, *c* (Å)	Angles α, β, γ (°)	Resolution (Å)
(*a*)	α-Amylase	*I*222	66.0, 140.9, 155.1	90, 90, 90	1.65
(*b*)	Albumin (BSA)	*C*222_1_	85, 125.5, 138.5	90, 90, 90	2.40
(*c*)	Aprotinin (BTI)	*P*2_1_2_1_2_1_	23, 28.7, 73.4	90, 90, 90	1.00
(*d*)	Catalase	*P*2_1_2_1_2_1_	68.7, 171.7, 192.8	90, 90, 90	1.51
(*e*)	Concanavalin A	*P*2_1_2_1_2_1_	66.8, 116.8, 123.1	90, 90, 90	1.39
(*f*)	Creatine kinase	*C*2	257.97, 68.2, 133.0	90, 114, 90	2.95
(*g*)	Glutathione *S*-transferase	*P*2_1_2_1_2_1_	90.4, 94.4, 113.6	90, 90, 90	2.60
(*h*)	Insulin	*I*2_1_3	78.6, 78.6, 78.6	90, 90, 90	1.15
(*i*)	Lysozyme	*P*4_3_2_1_2	77.4, 77.4, 38.0	90, 90, 90	1.16
(*j*)	Pyruvate kinase	*P*2_1_	141.5, 112.2, 170.4	90, 94, 90	2.70
(*k*)	Streptavidin	*P*2_1_	46.6, 85.8, 58.0	90, 98.9, 90	1.38
(*l*)	Thaumatin	*P*4_1_2_1_2	58.1, 58.1, 150.3	90, 90, 90	1.13
