# Supplementary material for: A generic cross-seeding approach to protein crystallization
Source: J Appl Crystallogr. 2025 Feb 17;58(Pt 2):383–91. doi: 10.1107/S1600576725000457 (PMC11957411; doi:10.1107/S1600576725000457)
Supplement: Supplementary file 1 [file j-58-00383-sup1.pdf]

JOURNAL OF APPLIED CRYSTALLOGRAPHY

Volume 58 (2025)

Supporting information for article:

A generic cross-seeding approach to protein crystallization

Ido Caspy, Shan Tang, Dom Bellini and Fabrice Gorrec

Notes: Crystallisation of the host proteins. Precipitate was systematically observed in the drops with diffraction-quality crystals of catalase, pyruvate kinase and thaumatin. Upon freezing, samples of creatine kinase and streptavidin did not yield crystals. Showers of microcrystals were frequently observed during crystallisation of insulin instead. The yield of BSA, BTI, GST and diffraction-quality crystals was typically very low (3-4 hits from 48 droplets), however results were improved with freshly prepared samples and seeding. For seeding (albumin, aprotinin and glutathione-S-transferase), 50 nL of seeding solution was initially added to the 1.5 µL crystallisation droplets. Seeding solutions were prepared with one drop containing relatively large diffraction-quality crystals (i.e. a ‘hit’) that was diluted in 80 µL of condition in the reservoir of an MRC plate (SWISSCI) for fragmentation with two cycles of two minutes on the high-speed oscillation mixer MXone (‘MixerDriveLevel’ default speed: 2500, SPT Labtech).

Table S1. The 12 commercial proteins used as host proteins.

	Protein	Origin (Merck)	MW (KDa)	Description
**a**	α-Amylase	A9857	53	amylose hydrolysase from Aspergillus oryzae
**b**	Albumin	05470	66.5	bovin serum albumin
**c**	Aprotinin	A6103	6.5	pancreatic trypsin inhibitor recombinant, expressed in Nicotiana (tobacco)
**d**	Catalase	C3155	248	tetrameric H2O2:H2O2 oxidoreductase from bovine liver
**e**	Concanavalin A	L7647	104-112	tetrameric lectin from Canavalia ensiformis (Jack bean)
**f**	Creatine kinase	10127566001	84	dimeric phosphokinase from rabbit muscle
**g**	Glutathione-S-Transferase	G6511	50	reduced dimeric redox agent from from equine liver
**h**	Insulin	I5500	5.8	hormone from bovine pancreas
**i**	Lysozyme	L6876	14.4	muramidase from hen egg white
**j**	Pyruvate kinase	P9136	237	tetrameric glycolysis enzyme from rabbit muscle
**k**	Streptavidin	S4762	53.6	tetrameric biotin-binding glycoprotein from Streptomyces avidinii
**l**	Thaumatin	T7638	22	sweetener from Thaumatococcus daniellii

Table S2. Results of initial crystallisation trials with RBBP9 and the cross-seeding mixture. The table lists the 16 conditions from the 96-condition MORPHEUS-FUSION screen (Molecular Dimensions) with which diffraction-quality crystals were produced when using the RBBP9 sample on its own (‘no addition’), when adding the stabilising solution (i.e. no fragments) or when adding the final cross-seeding mixture (integrating all the fragments) to the RBBP9 sample. Experiments were triplicated (a, b and c). The number 1 indicates a hit: a droplet that contained crystals large enough to be fished out readily and later showed good diffraction. The number 0 means no hit. A few droplets exhibiting showers of microcrystals were not listed. All the hits occurred within a few days.

Condition	no addition			stabilising solution			cross-seeding			formulation of the condition
	a	b	c	a	b	c	a	b	c	
A01	1	0	0	0	0	0	1	1	1	10% w/v PEG 20 000, 20% v/v PEG MME 500, 0.1 M MES/imidazole pH 6.5, 30 mM of each divalent cation 1, 20 mM of each alcohol
A05	1	1	1	1	0	0	0	0	1	10% w/v PEG 20 000, 20% v/v PEG MME 500, 0.1 M MOPS/HEPES-Na pH 7.5, 20 mM of each amino-acid, 20 mM of each alcohol
B04	1	1	0	0	1	1	0	0	0	10% w/v PEG 20 000, 20% v/v PEG MME 500, 0.1 M MES/imidazole pH 6.5, 20 mM of each carboxylic acid, 30 mM of each ethylene glycol
B08	0	0	0	1	1	0	0	0	0	10% w/v PEG 20 000, 20% v/v PEG MME 500, 0.1 M MOPS/HEPES-Na pH 7.5, 1 mM of each alkali, 30 mM of each ethylene glycol
B11	1	0	0	0	0	0	0	0	0	12.5% w/v PEG 1000, 12.5% w/v PEG 3350, 12.5% v/v MPD, 0.1 M MOPS/HEPES-Na pH 7.5, 10 mM of each polyamine, 30 mM of each ethylene glycol
C07	1	1	1	1	0	0	1	0	0	10% w/v PEG 20 000, 20% v/v PEG MME 500, 0.1 M MES/imidazole pH 6.5, 30 mM of each halide, 20 mM of each monosaccharide 1
D01	1	0	0	0	0	0	0	1	1	12.5% w/v PEG 1000, 12.5% w/v PEG 3350, 12.5% v/v MPD, 0.1 M MES/imidazole pH 6.5, 30 mM of each divalent cation 1, 20 mM of each monosaccharide 2
D05	0	0	0	0	0	0	1	1	1	12.5% w/v PEG 1000, 12.5% w/v PEG 3350, 12.5% v/v MPD, 0.1 M MOPS/HEPES-Na pH 7.5, 20 mM of each amino-acid, 20 mM of each monosaccharide 2
D10	1	1	1	1	1	1	0	1	0	10% w/v PEG 20 000, 20% v/v PEG MME 500, 0.1 M MES/imidazole pH 6.5, 0.3% of each vitamin, 20 mM of each monosaccharide 2
E10	0	0	0	0	1	1	0	1	1	10% w/v PEG 20 000, 20% v/v PEG MME 500, 0.1 M bicine/TRIS-base pH 8.5, 0.3% of each vitamin, 0.3% w/v of each cholic acid derivative
F05	1	1	1	1	1	1	1	1	1	10% w/v PEG 20 000, 20% v/v PEG MME 500, 0.1 M MES/imidazole pH 6.5, 20 mM of each amino-acid, 0.5% w/v of each cryo-polyol
F08	0	0	0	0	0	0	0	0	1	12.5% w/v PEG 1000, 12.5% w/v PEG 3350, 12.5% v/v MPD, 0.1 M MES/imidazole pH 6.5, 1 mM of each alkali, 0.5% w/v of each cryo-polyol
G08	1	0	0	1	1	0	0	1	0	10% w/v PEG 20 000, 20% v/v PEG MME 500, 0.1 M MES/imidazole pH 6.5, 1 mM of each alkali, 0.3% w/v of each NDSB
G11	0	0	0	0	0	0	1	1	1	12.5% w/v PEG 1000, 12.5% w/v PEG 3350, 12.5% v/v MPD, 0.1 M MES/imidazole pH 6.5, 10 mM of each polyamine, 0.3% w/v of each NDSB
G12	0	0	0	0	0	0	0	1	1	10% w/v PEG 20 000, 20% v/v PEG MME 500, 0.1 M MOPS/HEPES-Na pH 7.5, 0.2% w/v of each anaesthetic alkaloid, 0.3% w/v of each NDSB
H11	0	0	1	0	0	0	1	1	0	10% w/v PEG 20 000, 20% v/v PEG MME 500, 0.1 M MES/imidazole pH 6.5, 30 mM of each halide, 20 mM of each monosaccharide 1
**Total**			**19**			**16**			**25**	

Table S3. Crystallographic data summary table for the $P2_12_12_1$ crystal form of RBBP9 (PDB code: 9FCR). The coordinates of PDB 2QS9 were used as the search model for molecular replacement structure solution.

**Resolution range**	44.38 - 1.37 (1.42 - 1.37)
**Space group**	P 21 21 21
**Unit cell**	67.74 82.55 58.74 90 90 90
**Total reflections**	123660 (6678)
**Unique reflections**	65671 (4570)
**Multiplicity**	1.9 (1.5)
**Completeness (%)**	89.6 (35.7)
**Mean I/sigma(I)**	5.3 (0.3)
**Wilson B-factor**	18.2
**R-merge**	0.06 (0.95)
**R-meas.**	0.085 (1.35)
**R-pim**	0.06 (0.95)
**CC1/2**	0.99 (0.54)
**CC***	0.99 (0.84)
**Reflections used in refinement**	62574 (2447)
**Reflections used for R-free**	3077 (130)
**R-work**	0.20 (0.46)
**R-free**	0.23 (0.43)
**CC(work)**	0.97 (0.61)
**CC(free)**	0.94 (0.61)
**Number of non-hydrogen atoms**	3307
**Macromolecules**	2922
**Solvent**	385
**Protein residues**	366

Table S4. Results of crystallisation trials with RBBP9 and the 12 seeding solutions made from a single protein host (a-l, see proteins listed in Table S1). The MORPHEUS-FUSION condition D5 is 12.5 % w/v PEG 1000, 12.5 % w/v PEG 3350, 12.5 % v/v MPD, 0.1 M MOPS/HEPES-Na pH 7.5, 20 mM of each amino-acid, 20 mM of each monosaccharide 2; and the condition F8 is 12.5 % w/v PEG 1000, 12.5 % w/v PEG 3350, 12.5 % v/v MPD, 0.1 M MES/imidazole pH 6.5, 1 mM of each alkali, 0.5 % w/v of each cryo-polyol. For details on assessment procedure, see Table S2.

**Addition**	**Condition D5**			**Condition F8**		
	**a**	**b**	**c**	**a**	**b**	**c**
Seeds a	0	0	0	1	1	1
Seeds b	0	0	0	0	0	0
Seeds c	0	0	0	0	0	1
Seeds d	0	0	0	1	1	1
Seeds e	0	0	0	0	0	0
Seeds f	0	0	0	0	0	0
Seeds g	0	0	0	0	0	0
Seeds h	0	0	0	0	0	0
Seeds i	0	0	0	1	0	0
Seeds j	1	0	0	0	0	0
Seeds k	0	1	0	1	0	1
Seeds l	0	0	0	1	1	1
Mixture	1	1	1	0	0	1
None	0	0	0	0	0	0
Stabilising solution	0	0	0	0	0	0

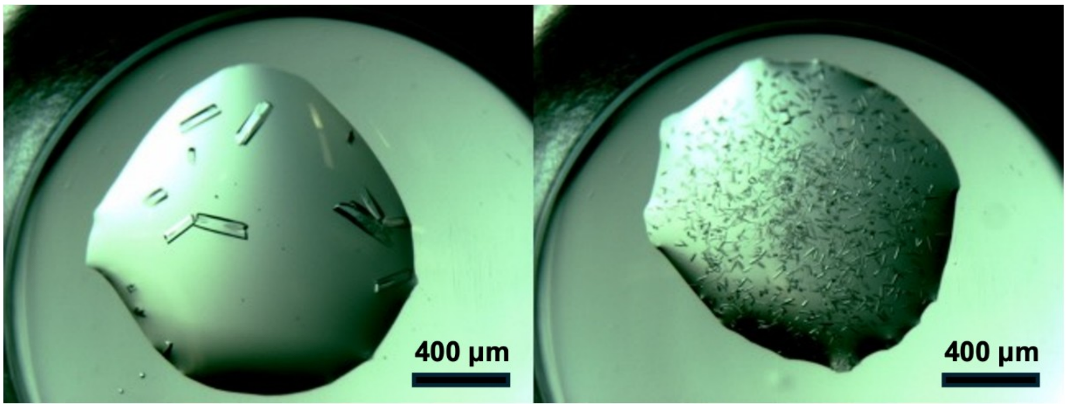

Figure S1. Initial homoepitaxial seeding assays (example of lysozyme). Adding the final cross-seeding mixture to samples used to produce the seeds resulted in substantial increases in nucleation sites compared to controls without seeds.

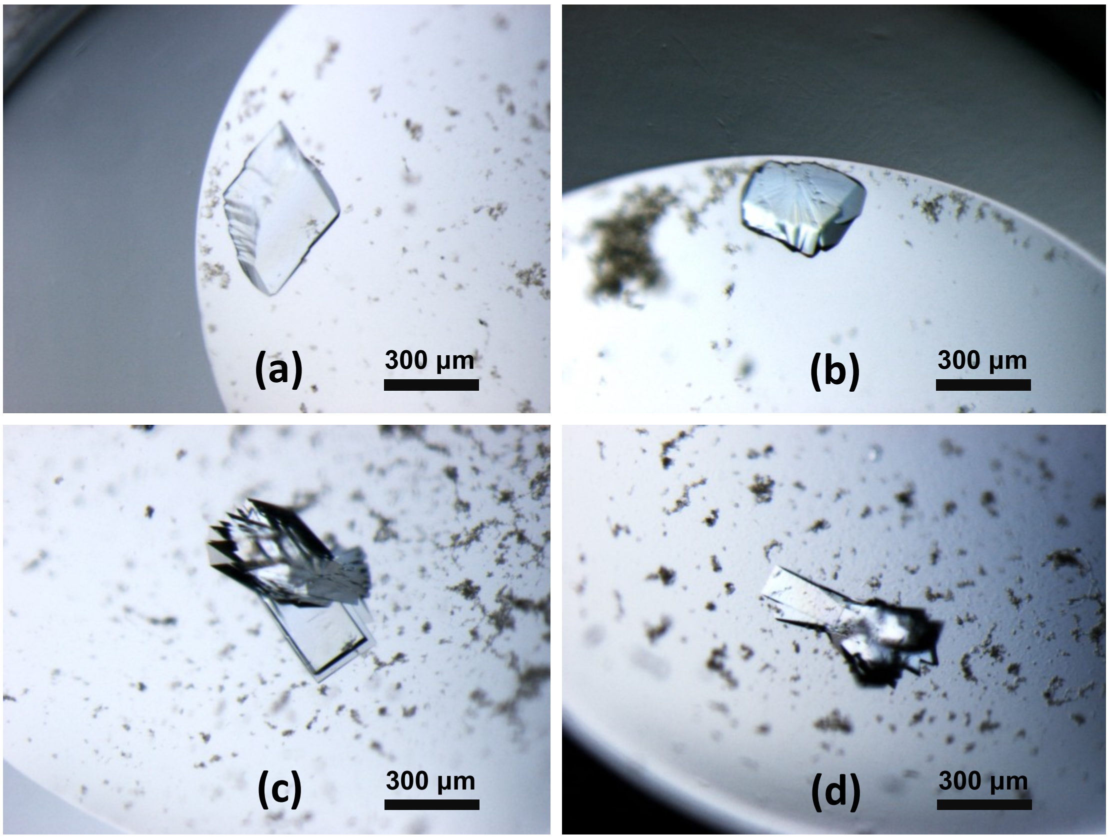

Figure S2. Light micrographs showing crystal hits listed in Table S4. The crystal form discovered in this study ($P2_12_12_1$) was reproduced with a) the cross-seeding mixture in condition D5 and b) the seeds made of α-amylase ("Seeds a") in condition F8. More typical bundles of rod-like shape crystals (space group $P2_1$) were also reproduced in condition F8, for example with c) the seeds made of lysozyme ("Seeds i") and d) the seeds made of thaumatin ("Seeds l") in condition F8.
